# Serum leptin and its relation to body composition, puberty, and metabolism in severe obesity

**DOI:** 10.1530/EC-25-0194

**Published:** 2025-07-16

**Authors:** Sabina Galiniak, Marek Biesiadecki, Rafał Podgórski, Natalia Dąbek, Ewa Gramatyka-Drążek, Ewelina Gaweł, Michael B Ranke, Bertram Flehmig, Martin Wabitsch, Stephanie Brandt, Elżbieta Petriczko, Małgorzata Wójcik, Ewa Małecka-Tendera, Mirosław Bik-Multanowski, Agnieszka Zachurzok, Artur Mazur

**Affiliations:** ^1^ Faculty of Medicine, Medical College, University of Rzeszów, Rzeszów, Poland; ^2^ Children’s Hospital, University of Tübingen, Tübingen, Germany; ^3^ Mediagnost GmbH, Reutlingen, Germany; ^4^ Division of Pediatric Endocrinology and Diabetes, Department of Pediatrics and Adolescent Medicine, Center for Rare Endocrine Diseases, Ulm, Germany; ^5^ Department of Pediatrics, Endocrinology, Diabetology, Metabolic Disorders and Cardiology of Developmental Age, Pomeranian Medical University, Szczecin, Poland; ^6^ Department of Pediatric and Adolescent Endocrinology, Pediatric Institute, Jagiellonian University Medical College, Cracow, Poland; ^7^ Department of Pediatrics and Pediatric Endocrinology, Medical University of Silesia, School of Medicine in Katowice, Katowice, Poland; ^8^ Department of Medical Genetics, Jagiellonian University Medical College, Cracow, Poland; ^9^ Institute of Human Genetics, University Hospital, LMU Munich, Munich, Germany; ^10^ Department of Pediatrics, Faculty of Medical Sciences in Zabrze, Medical University of Silesia, Zabrze, Poland

**Keywords:** severe obesity, leptin, biologically active leptin, body composition, lipid profile

## Abstract

Childhood severe obesity (SO) is a growing public health concern. Leptin (LEP) and its biologically active fraction (BioLEP) play a key role in energy balance and body mass regulation. Understanding their relationship with anthropometric and metabolic parameters is crucial for improving SO treatment. This study analyzed total LEP, BioLEP, and the BioLEP/LEP ratio in children with SO and their associations with body composition, lipid profile, and pubertal development. The study included 461 children (245 girls and 216 boys) aged 0–19 years with SO. Anthropometric parameters, metabolic indices, and pubertal stages (Tanner scale) were assessed. LEP and BioLEP concentrations were measured using ELISA, and the BioLEP/LEP ratio was calculated. The median LEP concentration was 56.53 ng/mL, and BioLEP was 53.66 ng/mL, with a BioLEP/LEP ratio of 0.94. Girls had significantly higher LEP (64.40 vs 49.66 ng/mL, *P* < 0.001) and BioLEP (59.90 vs 46.93 ng/mL, *P* < 0.001) than boys. LEP and BioLEP correlated positively with BMI, waist and hip circumference, and fat mass percentage, while negatively with waist-to-hip ratio and fat-free mass. The BioLEP/LEP ratio correlated positively with insulin and lipid profile parameters (total cholesterol, HDL, and triglycerides). LEP and BioLEP play a significant role in SO, particularly in fat mass regulation. The BioLEP/LEP ratio is more closely linked to lipid metabolism. These findings provide insights into the hormonal and metabolic profile of SO, supporting targeted diagnostic and therapeutic approaches.

## Introduction

Childhood obesity, particularly severe obesity (SO), is a growing global health concern. Its prevalence has risen dramatically over recent decades, with significant implications for public health and individual well-being (1, 2). SO in children is linked to a higher risk of metabolic disorders, such as type 2 diabetes, abnormal lipid levels, and cardiovascular conditions, as well as long-term persistence of these disorders into adulthood (3, 4). SO is a complex condition with a multifactorial etiology that often involves a significant genetic and hormonal component. Unlike common forms of obesity primarily driven by environmental factors, SO frequently results from underlying genetic mutations or hormonal imbalances that disrupt the physiological mechanisms regulating energy balance, appetite, and metabolism (5, 6). Understanding the biological mechanisms underlying SO is critical for identifying potential diagnostic markers and therapeutic targets.

Leptin (LEP), a hormone primarily secreted by adipose tissue, plays a pivotal role in regulating energy homeostasis and body weight. It acts through hypothalamic receptors to suppress appetite and increase energy expenditure (7). In states of obesity, circulating LEP concentrations are often elevated, reflecting the increased adipose tissue mass, but this hyperleptinemia is commonly accompanied by LEP resistance, where its physiological effects are diminished (8, 9). Beyond total LEP concentration, recent research highlights the importance of biologically active leptin (BioLEP), which represents the fraction of LEP capable of binding to its receptors and exerting its biological effects (10). The ratio of BioLEP to total LEP (BioLEP/LEP) provides additional insight into LEP functionality and may help elucidate the pathophysiology of obesity.

In children with SO, the relationship between total LEP, BioLEP, and clinical parameters remains insufficiently explored. Identifying how these markers correlate with anthropometric, metabolic, and pubertal factors can provide a deeper understanding of the hormonal dysregulation that characterizes this condition. Such insights are particularly important for younger populations, where early interventions may prevent the progression of obesity and its complications.

This study aims to investigate the levels of total LEP and BioLEP in children with SO. By analyzing their relationship with body composition, lipid profile, and pubertal status, we seek to uncover patterns that could inform clinical management and contribute to the development of targeted therapies for this vulnerable population.

## Subjects and methods

The study forms part of a prospective clinical investigation conducted at four specialized medical centers in Poland: Rzeszów, Kraków, Zabrze, and Szczecin. It aimed to include a total of 500 participants aged 0–19 years, all characterized by SO, excessive appetite, and compulsive food-seeking behaviors (11). The patients were enrolled from both inpatient and outpatient departments of four pediatric endocrinology medical centers between May 2022 and May 2024. Of the planned 500 samples from participants, 461 were collected. The missing samples were due to either a lack of consent for blood collection or difficulties in cooperation between the child and the medical staff.

### Study participants

The research sample included 461 children and adolescents (216 boys and 245 girls) who met the study criteria. Inclusion criteria and exclusion criteria were as previously described (12). In brief, participants were between 0 and 19 years old and had SO, defined by age-specific BMI thresholds (over 24 kg/m^2^ for children under 2, over 30 kg/m^2^ for those aged 2–6, over 35 kg/m^2^ for ages 6–14, and over 40 kg/m^2^ for those older than 14), or documented SO in the last 6 months. They exhibited hyperphagia and food-seeking behavior, with the appropriate questionnaires completed by the participants (if over 8 years old) or their caregivers (if under 8), and all caregivers completed the Hyperphagia Questionnaire for Clinical Trials. Participants were excluded if they did not provide the required written informed consent or if they had a secondary cause of obesity, such as a previously diagnosed genetic syndrome or current treatment with medications that cause weight gain.

Data on birth weight and age at onset of obesity were collected from available medical records.

Anthropometric assessment (height, weight, and waist and hip circumference) was performed in the study participants as reported previously (4). Waist-to-hip ratio (WTH) was calculated. Bioimpedance analysis was performed using the TANITA MC-580 M S MDD, TANITA MC-780MA-N, and TANITA MC-780 P MA devices. The Tanner scale was used to assess pubertal development in 447 children. In 14 children, developmental assessment could not be performed due to lack of consent or difficulties in cooperation.

### Biochemical analyses

Venous peripheral blood was collected into tubes containing a clotting activator. After clotting, the samples were centrifuged, and the resulting serum was transferred to separate tubes and cryopreserved until hormone analysis. The samples were thawed only once, on the day of the LEP and BioLEP assays. Serum biochemical analyses (fasting insulin, total cholesterol, high-density lipoprotein – HDL, and low-density lipoprotein – LDL) were performed by standard methods.

### LEP and BioLEP assay

The total LEP concentration was quantified using a sandwich enzyme-linked immunosorbent assay (ELISA) (E077, Mediagnost, Germany). The LEP assay uses monoclonal antibodies directed toward nonspecific epitopes of LEP. BioLEP was measured using an ELISA (L07, Mediagnost, Germany), where the analyte was captured by a recombinant leptin receptor immobilized on a microtiter plate. The BioLEP assay selects LEP which contains the epitopes relevant for binding to the LEP receptor, thus biologically active LEP.

All procedures were carried out following the manufacturer’s instructions. According to the manufacturer, the inter- and intra-assay coefficients of variation for both ELISAs are below 10%. The analytical sensitivity of the assays was below 0.01 ng/mL for both tests. Hormone determinations were carried out in duplicate. Absorbance measurements were conducted using a Tecan Infinite 200 PRO multimode reader (Tecan Group Ltd, Switzerland). The ratios of BioLEP to total LEP (BioLEP/LEP) were calculated.

### Ethical approval

The study protocol was approved by the Bioethics Committee of the Medical University of Silesia (PCN/CBN/0022/KB1/137/I/21/22, date: February 8, 2022) and received favorable opinions from the local bioethics committees of all participating centers. Written informed consent was obtained from the parents or guardians of the patients, as well as from patients aged 13 years or older. All procedures involving human participants followed the ethical standards of institutional and national research committees and complied with the Declaration of Helsinki (1964) and its subsequent amendments or equivalent ethical guidelines.

### Statistical analysis

All statistical analyses were conducted using the STATISTICA software package (StatSoft Inc., USA). Data are presented as median and interquartile range (IQR). The Shapiro–Wilk test was used to assess the normality of the data distribution, revealing that most variables did not follow a normal distribution. Consequently, non-parametric tests were applied. The Mann–Whitney test was used for comparisons, Spearman’s correlation was used to assess the correlations, and a *P*-value of less than 0.05 was considered statistically significant.

## Results

The study cohort consisted of 461 participants, with 245 girls and 216 boys, analyzed across the total group, sex, and pubertal development (puberty vs prepuberty). A total of 382 children had entered puberty, as determined by reaching at least Tanner stage II in one assessed parameter, while 65 remained in the prepubertal phase, classified as Tanner stage I.

Key differences were observed in various anthropometric and body composition parameters (Table 1).

**Table 1:** Basic characteristics of the study group*.

Whole study cohort	Available data	Overall	Sex			Pubertal development		
			Girls (*n* = 245)	Boys (*n* = 216)	*P*	Prepubertal (*n* = 65)	Pubertal (*n* = 382)	*P*
Age (years)	461	13.92 (12, 15)	14 (12, 15.8)	13 (11.75, 15)	0.057	7.5 (6, 10)	14 (12.8, 15.8)	<0.001
Birth weight (g)	450	3,459.5 (3,070, 3,820)	3,360 (3,010, 3,660)	3,580 (3,180, 4,000)	<0.001	3,450 (3,140, 3,870)	3,454.5 (3,045, 3,805)	0.370
Age at onset of obesity (years)	445	4 (1.5, 8)	4 (2, 8)	4 (1, 8)	0.814	2 (1, 5)	4.5 (2, 8)	<0.001
Height (cm)	461	166.3 (158.4, 173)	163.5 (157, 168)	171 (162.75, 176.05)	<0.001	138 (123, 152)	167.5 (162, 174)	<0.001
Weight (kg)	461	109.5 (95, 125)	106 (93.7, 119)	114 (97.6, 131.75)	<0.001	70.4 (49, 86)	113 (103, 127)	<0.001
BMI (kg/m^2^)	461	39.41 (36.22, 42.55)	40.06 (35.91, 43.06)	39.04 (36.44, 42.12)	0.544	35.28 (31.04, 38.16)	40.23 (36.9, 42.98)	<0.001
BMI z-score	453	2.81 (2.57, 3.52)	2.73 (2.48, 3.47)	2.85 (2.67, 3.56)	<0.001	3.14 (2.73, 4.45)	2.77 (2.55, 3.45)	<0.001
Waist circumference (cm)	454	112 (102, 121)	108 (100, 118)	115.75 (107.75, 123.25)	<0.001	96 (83, 108)	114 (104, 123)	<0.001
Hip circumference (cm)	453	123.5 (115, 131)	125 (116, 133)	122 (113, 130)	0.029	104 (88, 113)	125 (119.5, 133)	<0.001
WTH	452	0.914 (0.857, 0.976)	0.879 (0.828, 0.936)	0.956 (0.899, 1.018)	<0.001	0.953 (0.898, 1.007)	0.904 (0.851, 0.969)	<0.001
Fat mass (kg)	372	50.35 (43.25, 59.65)	50.9 (43, 63.3)	49.9 (44.1, 57.9)	0.407	37.4 (25.13, 49.3)	51.7 (45.1, 61.2)	<0.001
Fat mass (%)	371	46.1 (41.5, 50.7)	47.85 (43.5, 51.9)	43.5 (39.6, 49)	<0.001	48.8 (44.7, 52)	45.7 (41.3, 50.6)	0.021
Fat-free mass (kg)	371	58.7 (52.1, 68.3)	56.5 (50, 61.8)	64.05 (55.5, 75.15)	<0.001	37.15 (31.4, 42.55)	59.85 (54.7, 69.8)	<0.001
Fat-free mass (%)	374	53.95 (49.4, 58.7)	52.2 (48.1, 56.5)	56.5 (51, 60.6)	<0.001	51.35 (48.2, 56.75)	54.3 (49.4, 58.7)	0.133
Insulin (IU/mL)	445	25.4 (18.11, 36.08)	24.51 (17.3, 33.6)	26.25 (18.37, 37.65)	0.238	19 (13.19, 31.07)	25.9 (18.6, 37.1)	0.002
Cholesterol (mg/dL)	451	147 (5.68, 177)	146.36 (5.64, 175)	149 (5.73, 180)	0.653	155 (94.36, 184)	146.36 (5.37, 175)	0.174
HDL (mg/dL)	451	37 (1.43, 44.1)	38.1 (1.45, 45.5)	36.2 (1.42, 43.4)	0.255	39.75 (26.15, 47.27)	37 (1.39, 44)	0.183
LDL (mg/dL)	450	83.82 (3.41, 110.35)	84.45 (3.4, 109)	82.54 (3.46, 111.9)	0.863	92 (66, 123.5)	82 (3.3, 109)	0.046
Triglycerides (mg/dL)	449	98 (2.18, 143)	95.6 (2.08, 137)	99.53 (2.56, 154)	0.111	98 (52.2, 131)	97.68 (2.05, 144)	0.966

* Data are presented as median and IQR in parentheses.

The median age of the cohort was 13.92 years (IQR: 12–15), with no significant sex difference (*P* = 0.057). Boys were taller (median: 171 vs 163.5 cm; *P* < 0.001) and had a higher weight (median: 114 vs 106 kg; *P* < 0.001) than girls. Boys had a higher WTH ratio than girls (*P* < 0.001).

BMI did not differ significantly between sexes (*P* = 0.544), but it was higher in the pubertal group compared to the prepubertal group (*P* < 0.001). Boys had significantly higher fat-free mass (median: 64.05 vs 56.5 kg; *P* < 0.001) and a lower fat mass percentage (median: 43.5 vs 47.85%; *P* < 0.001) compared to girls. Fat-free mass was also higher in the pubertal group (median: 59.85 vs 37.15 kg; *P* < 0.001), while fat mass percentage was lower in this group (median: 45.7 vs 48.8%; *P* = 0.021). No significant differences in absolute fat mass were observed between sexes (*P* = 0.407), but fat mass was higher in the pubertal group compared to the prepubertal group (*P* < 0.001). Insulin levels were similar in boys and girls but were statistically higher in pubertal children than in prepubertal children (*P* = 0.002).

No significant sex differences were found in total cholesterol, HDL, or triglycerides. LDL cholesterol did not differ by sex (*P* = 0.863) but was significantly higher in the prepubertal group (median: 92 mg/dL vs 82 mg/dL; *P* = 0.046). No other significant differences in lipid parameters were observed between the pubertal and prepubertal groups.

**Figure 1:**
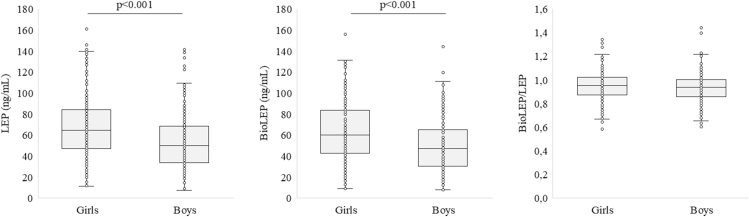
The levels of LEP, BioLEP, and the BioLEP/LEP ratio in the study group depending on sex.

The analysis of LEP and BioLEP levels in the entire study group revealed the following results (Fig. 1). The median LEP concentration was 56.53 ng/mL, with a range of 7.32–162.51 ng/mL (IQR: 39.51–77.05 ng/mL). The median concentration of BioLEP was slightly lower at 53.66 ng/mL, with a range of 7.80–155.93 ng/mL (IQR: 35.84–75.75 ng/mL). The ratio of BioLEP/LEP had a median value of 0.94, ranging from 0.58 to 1.44 (IQR: 0.863–1.015).

**Figure 2:**
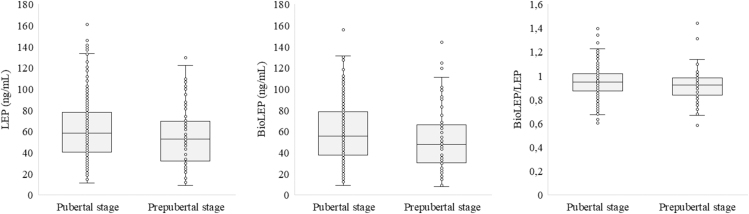
The levels of LEP, BioLEP, and the BioLEP/LEP ratio in the study group depending on pubertal stage.

Figures 1 and 2 present the levels of LEP, BioLEP, and the BioLEP/LEP ratio in the study group, stratified by sex and pubertal stage.

Girls exhibited higher median LEP levels (64.40 ng/mL; IQR: 47.34–84.09) compared to boys (49.66 ng/mL; IQR: 33.81–68.59, *P* < 0.001). The range of LEP levels was also wider in girls (11.46–162.51 ng/mL) than in boys (7.32–141.07 ng/mL). Similarly, the median BioLEP level was higher in girls (59.90 ng/mL; IQR: 42.66–83.45) compared to boys (46.93 ng/mL; IQR: 30.02–64.79, *P* < 0.001). The range of BioLEP levels in girls (8.92–155.93 ng/mL) exceeded that observed in boys (7.80–143.88 ng/mL). In contrast, the BioLEP/LEP ratio showed less variation between sexes. There was no sex difference in the BioLEP/LEP ratio (girls: 0.95; IQR: 0.87–1.02, boys: 0.94; IQR: 0.86–1.00). However, the range of the ratio was broader in boys (0.60–1.44) than in girls (0.58–1.34).

In the prepubertal stage, the median LEP level was 52.48 ng/mL (IQR: 33.59–69.22), with a range from 8.98 to 129.39 ng/mL. In contrast, in patients categorized as pubertal, the median LEP level was higher at 58.17 ng/mL (IQR: 40.60–77.90), with a broader range of 11.46–162.51 ng/mL (*P* = 0.160). Similarly, the median BioLEP level increased from 47.75 ng/mL (IQR: 31.03–65.47; range: 7.80–143.88 ng/mL) in the prepubertal stage to 55.42 ng/mL (IQR: 37.51–78.20; range: 8.92–155.93 ng/mL) in the pubertal stage (*P* = 0.068).

The BioLEP/LEP ratio showed no difference between prepubertal and pubertal categorized patients (*P* = 0.098).

**Figure 3:**
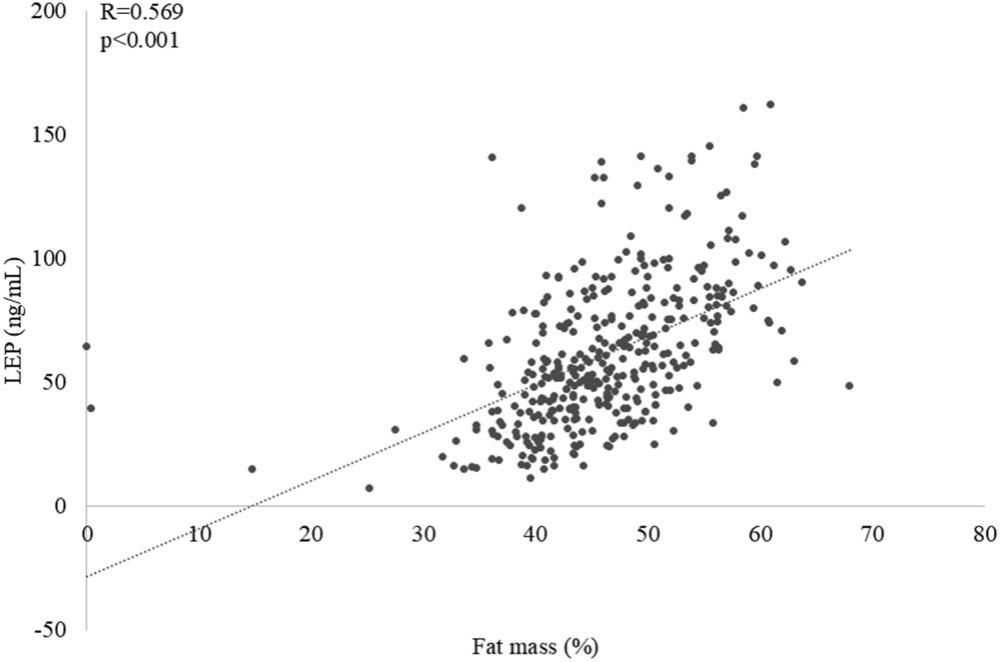
The relationship between LEP and BioLEP concentration in the study group.

The concentrations of LEP and BioLEP were positively and strongly correlated (Fig. 3). There was no correlation between LEP concentration and the BioLEP/LEP ratio (*R* = 0.03, *P* = 0.513). However, a weak correlation was observed between BioLEP and the BioLEP/LEP ratio (*R* = 0.267, *P* < 0.001).

The analysis revealed several significant correlations between the studied variables and the LEP, BioLEP, and BioLEP/LEP indices (Table 2). Both LEP and BioLEP exhibited weak but statistically significant positive correlations with age (*R* = 0.148 and *R* = 0.159, respectively; *P* = 0.001). Similarly, the age of obesity onset showed weak but significant positive correlations with BioLEP (*R* = 0.104; *P* = 0.028) and BioLEP/LEP (*R* = 0.097; *P* = 0.040).

**Table 2:** Spearman’s rank correlation coefficients and *P*-values.

	LEP		BioLEP		BioLEP/LEP	
	*R*	*P*	*R*	*P*	*R*	*P*
Age (years)	0.148	0.001	0.159	0.001	0.066	0.155
Birth weight (g)	−0.016	0.738	−0.021	0.660	−0.006	0.900
Age at onset of obesity (years)	0.083	0.081	0.104	0.028	0.097	0.040
Height (cm)	−0.050	0.289	−0.056	0.234	−0.039	0.404
Weight (kg)	0.247	<0.001	0.243	<0.001	0.018	0.692
BMI (kg/m^2^)	0.449	<0.001	0.459	<0.001	0.106	0.023
BMI z-score	0.043	0.362	−0.030	0.521	−0.254	<0.001
Waist circumference (cm)	0.248	<0.001	0.249	<0.001	0.032	0.496
Hip circumference (cm)	0.420	<0.001	0.414	<0.001	0.061	0.196
WTH	−0.162	<0.001	−0.157	<0.001	−0.031	0.515
Fat mass (kg)	0.484	<0.001	0.500	<0.001	0.100	0.054
Fat mass (%)	0.569	<0.001	0.576	<0.001	0.083	0.110
Fat-free mass (kg)	−0.125	0.016	−0.129	0.013	−0.056	0.283
Fat-free mass (%)	−0.570	<0.001	−0.579	<0.001	−0.085	0.101
Insulin (IU/mL)	0.157	<0.001	0.159	<0.001	0.026	0.59
Cholesterol (mg/dL)	−0.022	0.638	0.020	0.665	0.178	<0.001
HDL (mg/dL)	0.082	0.082	0.130	0.006	0.230	<0.001
LDL (mg/dL)	−0.034	0.468	0.004	0.931	0.161	0.001
Triglycerides (mg/dL)	−0.107	0.023	−0.063	0.180	0.168	<0.001

Weak positive correlations were observed between LEP and BioLEP indices and anthropometric parameters, including body weight (*R* = 0.247 and *R* = 0.243; *P* < 0.001), BMI (*R* = 0.449 and *R* = 0.459; *P* < 0.001), waist circumference (*R* = 0.248 and *R* = 0.249; *P* < 0.001), and hip circumference (*R* = 0.420 and *R* = 0.414; *P* < 0.001). Surprisingly, we found negative correlations between LEP and BioLEP concentrations and WTH ratio (*R* = −0.162 and *R* = −0.157; *P* < 0.001). There was no correlation between the concentration of the studied adipokines, BioLEP/LEP, and the birth weight of children with SO.

**Figure 4:**
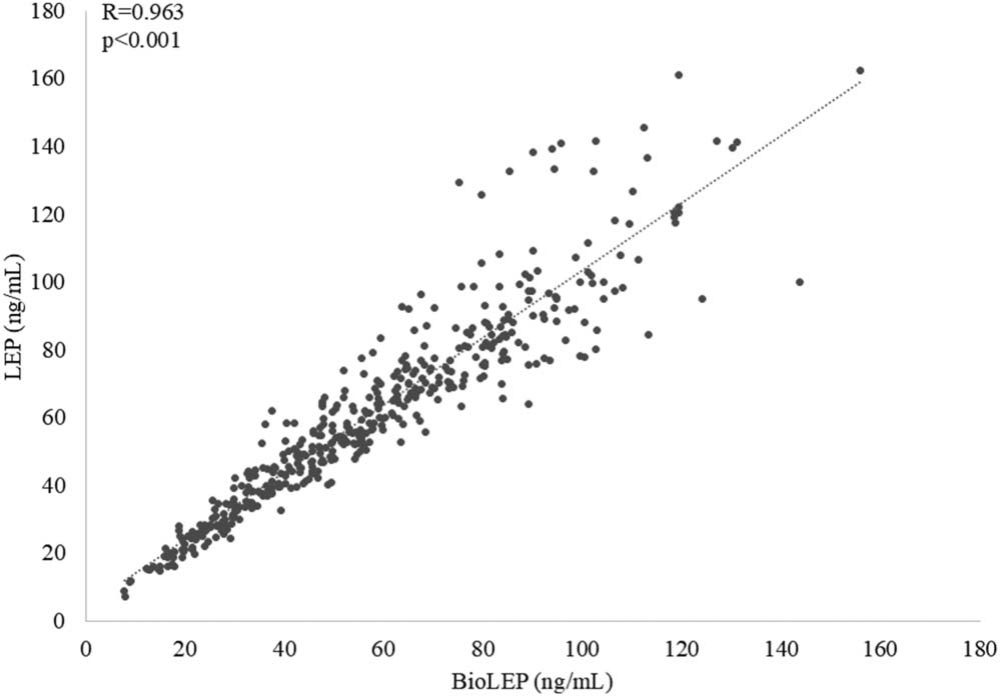
The relationship between LEP concentration and fat mass in children and adolescents with SO.

Furthermore, a strong positive association was observed between the percentage of fat mass and both LEP (*R* = 0.569; *P* < 0.001, Fig. 4.) and BioLEP (*R* = 0.576; *P* < 0.001). Conversely, the percentage of fat-free mass was strongly negatively correlated with LEP (*R* = −0.570; *P* < 0.001) and BioLEP (*R* = −0.579; *P* < 0.001). Moreover, LEP and BioLEP correlated positively with insulin (*R* = 0.157 and *R* = 0.159; *P* < 0.001).

The BioLEP/LEP index showed significant positive correlations with lipid profile parameters, including total cholesterol (*R* = 0.178; *P* < 0.001), HDL (*R* = 0.230; *P* < 0.001), and LDL (*R* = 0.161; *P* = 0.001). In addition, triglycerides positively correlated with BioLEP/LEP (*R* = 0.168; *P* < 0.001) and negatively with LEP (*R* = −0.107; *P* = 0.023).

## Discussion

This study aimed to characterize the levels of total LEP and BioLEP, and to assess the utility of the BioLEP/LEP ratio in a large cohort of children with SO. Our findings revealed strong associations between LEP and BioLEP concentrations and anthropometric indices and metabolic parameters, suggesting that both total and bioactive LEP may reflect key elements of adiposity and metabolic health in this high-risk population.

The median concentration of LEP was 56.53 ng/mL in our study cohort with SO. Similarly, in the study of Leoni *et al.*, plasma LEP concentration was 48.45 ± 41.49 ng/mL in 47 children with obesity (13). In 77 Polish children with obesity, the LEP concentration was 40.5 ng/mL (14). These differences may reflect variations in obesity severity, age distribution, pubertal status, and assay methods across studies. Notably, our cohort was composed exclusively of individuals with SO, which may explain the comparatively higher leptin levels.

In this study, the median BioLEP concentration was 53.66 ng/mL, while BioLEP/LEP had a median value of 0.94. Serum BioLEP concentrations may be lower than LEP concentrations, depending on the degree of leptin resistance and the efficiency of its conversion in the bodies of children with obesity. On the other hand, the mean levels of BioLEP and LEP were almost identical (41.1 ± 25.2 vs 41.1 ± 25.4 ng/mL) in 70 children and adolescents with SO (15). Similar to our study, a wide range of LEP and BioLEP concentrations was observed in the study by Stanik *et al.* A BioLEP/LEP ratio close to 1, as observed in our study, suggests that a high proportion of the circulating LEP in children with SO is biologically active. This finding is particularly noteworthy because it indicates that the total LEP concentration largely reflects the functional LEP levels in this population. In the study by Stanik *et al.*, BioLEP and LEP levels were highly concordant, with a ratio of 100.7 ± 6.1% (15). However, the variability in the ratio (range: 0.58–1.44) points to individual differences in LEP bioactivity, which could stem from various factors, such as genetic mutations, post-transcriptional regulation of LEP, or differences in receptor binding efficiency (10, 16, 17, 18). The ratio BioLEP/LEP should be 1.0 under theoretical conditions. However, under real measurement conditions, there is always a certain variability. Thus, a ratio range of 0.7–1.3 is probably due to methodological circumstances, including assay sensitivity or calibration.

Our results demonstrate that girls with SO exhibit significantly higher levels of both LEP and BioLEP compared to boys, which is consistent with previous research indicating that adiposity and LEP levels are typically higher in females, particularly during puberty (19, 20). This sexual dimorphism may be partially explained by hormonal influences, including estrogen, which is known to upregulate LEP production (21, 22). The higher LEP levels in girls may also reflect greater fat mass accumulation, as observed in this study. On the other hand, no differences in LEP and BioLEP concentrations between the sex of the study participants were observed by Stanik *et al.* (15). Despite these differences, the BioLEP/LEP ratio remained relatively stable across sexes, suggesting comparable LEP bioactivity regardless of sex. This finding is significant, as it implies that sex differences in LEP levels are primarily quantitative rather than qualitative in nature.

There was no difference in LEP, BioLEP, and BioLEP/LEP concentrations in children depending on puberty. Nevertheless, leptin’s role in initiating puberty is well documented, and its elevated levels in pubertal children most probably reflect the physiological demands of growth and maturation (23). However, the slight increase in the BioLEP/LEP ratio during puberty compared to prepuberty may indicate improved LEP functionality, potentially contributing to the observed hormonal changes during this developmental period.

The significant positive correlations between LEP and BioLEP with anthropometric measures, including body weight, BMI, and fat mass percentage, confirm the role of LEP as a biomarker of adiposity. The plasma level of LEP was also found to correlate with age among children with obesity in Italy (13). Notably, our study found no association between LEP, BioLEP, and BMI z-score, which contrasts with the findings of Leoni *et al.*, who reported a significant positive correlation (*r* = 0.36, *P* < 0.001) (13). Similar to the previous report of Niklovitz *et al.* (24), in our population, BioLEP was slightly more associated with BMI and fat mass compared to LEP. The strong associations with fat mass percentage and the inverse correlations with fat-free mass percentage highlight leptin’s primary role in reflecting adipose tissue stores. These findings align with previous studies suggesting that LEP secretion is directly proportional to the amount of fat mass (25, 26). Interestingly, the significant correlations between LEP levels and hip and waist circumferences underscore its potential utility as an indicator of both overall and central adiposity. Similarly, positive correlations between serum LEP and waist circumference (*r* = 0.6) and WTH ratio (*r* = 0.48) were observed in individuals with obesity (21). Surprisingly, in our study, we found a negative correlation between LEP, BioLEP, and WTH ratio in children with SO. It seems that both the adiposity location and the amount of adipocyte tissue may be determinants of circulating leptin. Visceral fat, which predominates at higher WTH, has different metabolic properties and may produce less leptin compared to subcutaneous fat (27). This could explain the lower LEP and BioLEP concentrations in groups with higher WTH. It should also be noted that the BioLEP/LEP ratio showed weaker correlations with anthropometric and fat mass indices compared to LEP and BioLEP alone, despite reaching statistical significance. This attenuated association may be partially due to the mathematical nature of ratio variables, which can introduce additional variability and reduce sensitivity to underlying biological relationships.

The lack of significant correlation between LEP, BioLEP, and the BioLEP/LEP ratio with birth weight suggests that birth weight does not directly influence leptin levels in this study population. However, another study has reported that low birth weight is associated with increased LEP levels during childhood (28). Moreover, a study published by Shekhawat *et al.* found that neonatal cord LEP concentrations correlate with birth weight, suggesting a relationship between these factors (29). These discrepancies may be due to differences in study populations, methodologies, or the timing of leptin measurements. Furthermore, our results reveal that the age at onset of obesity appears to be more strongly associated with BioLEP levels, which could indicate the importance of BioLEP in the early stages of obesity development. The BioLEP/LEP ratio also showed a correlation with age at obesity onset, which further supports the idea that the balance between BioLEP and LEP may be related to the timing of obesity development. Furthermore, the observed positive correlations between LEP, BioLEP, and fasting insulin levels reinforce the role of leptin as a marker of insulin resistance in pediatric obesity, a relationship well documented in the literature (30, 31).

The BioLEP/LEP ratio, often considered a marker of LEP sensitivity, exhibited significant correlations with lipid profile parameters, including total cholesterol, HDL, LDL, and triglycerides. This suggests a potential link between LEP bioactivity and metabolic health. The positive correlation with HDL, often regarded as protective against cardiovascular disease, raises intriguing questions about the functional role of BioLEP in lipid metabolism. Conversely, the negative correlation between LEP levels and triglycerides may reflect the complex interplay between LEP resistance and metabolic dysregulation in SO. Nevertheless, a positive correlation was observed previously between serum LEP and cholesterol, triglycerides, and LDL in children with obesity (21). High LEP levels in children with obesity, and their dependence on adipose tissue content, may suggest the presence of leptin resistance related to an altered leptin receptor/post-receptor pathway in this group (14).

The distinct associations between LEP, BioLEP, and their ratio with anthropometric and metabolic parameters have important clinical implications. The stability of the BioLEP/LEP ratio across sexes and pubertal stages suggests that it may serve as a robust biomarker of LEP bioactivity. This could be particularly valuable in identifying children with SO who may benefit from targeted interventions aimed at improving LEP sensitivity, such as lifestyle modifications or pharmacological intervention. Furthermore, the strong correlation of LEP indices with fat mass highlights the potential utility of these measures in monitoring adiposity and treatment outcomes in pediatric obesity management. Consistent with our findings, Benbaibeche *et al.* reported that elevated serum leptin levels may serve as a predictive marker for metabolic risk in children and adolescents with obesity, highlighting its potential utility in the identification of uncontrolled eating and early risk stratification (32). A recent systematic review confirmed that leptin is one of the most consistently elevated adipokines in pediatric obesity and may serve as a reliable biomarker for early identification of metabolic dysfunction (33).

While this study provides valuable insights, several limitations should be noted. First, the cross-sectional design precludes causal inferences regarding the relationships between LEP indices, anthropometric measures, and metabolic parameters. Longitudinal studies are required to gain a deeper understanding of the dynamic changes in LEP bioactivity over time and their impact on obesity progression and associated complications. Second, although our sample size was sufficient to detect statistically significant differences and correlations, we did not perform an *a priori* power analysis. Future prospective studies should include power calculations to ensure sufficient sensitivity for detecting clinically meaningful effects and to validate our findings across more diverse pediatric populations.

Moreover, the study focused exclusively on children with SO, limiting the generalizability of findings to children with less SO or those without early-onset disease. Another limitation pertains to external validity. The study population was recruited from four specialized pediatric endocrinology centers, and although this allowed for detailed clinical assessment, it may introduce selection bias and limit the generalizability of the findings to other populations. Importantly, while this article does not include lifestyle-related variables, such as dietary intake, physical activity levels, or socioeconomic background, these data were collected as part of the larger research project and will be analyzed in subsequent publications. Future studies integrating these variables will allow for a more comprehensive assessment of factors influencing leptin biology in pediatric obesity. Finally, as this study was cross-sectional, it does not include follow-up data on the effects of lifestyle or pharmacological interventions on LEP and BioLEP levels.

Another limitation of the study is the lack of a control group; however, our study design focuses solely on children with SO in Poland. Future research should explore these relationships in more diverse pediatric populations.

This study introduces several novel findings in the field of childhood SO. One of the key contributions is the identification of the BioLEP/LEP ratio as a potential metabolic marker, particularly in relation to lipid metabolism, which has been less explored in pediatric populations. In addition, with a cohort of 461 children, this study is one of the largest investigations of LEP and BioLEP in children with SO, providing robust data on their role in energy balance and pubertal development. Furthermore, while the role of LEP in obesity is well established, this study provides new evidence linking the BioLEP/LEP ratio more closely to lipid metabolism, suggesting its potential as a biomarker for metabolic risk in SO. Importantly, while our findings reveal meaningful associations between LEP indices and both adiposity and metabolic parameters, they should be interpreted as exploratory due to the observational nature of the study and do not imply causality.

## Conclusion

In conclusion, our findings underscore the association of LEP and its bioactive fraction in the pathophysiology of SO. By elucidating the relationships between LEP indices, body composition, and metabolic health, this study may contribute to the identification of candidate markers for future targeted interventions. These results should be interpreted in the context of the study’s cross-sectional design, and further longitudinal studies are needed to confirm these associations and explore causal mechanisms.

## Declaration of interest

The authors declare that there is no conflict of interest that could be perceived as prejudicing the impartiality of the work reported.

## Funding

This work was funded in whole by the National Science Centre, Poland (2021/41/B/NZ5/01676).

## Author contribution statement

The study was designed by MB-R, BF, MW, EP, MW, EM-T, MB-M, AZ, and AM. The study was conducted by SG and RP. Data were collected by SG, ND, EG-D, EG, EP, MW, EM-T, MB-M, AZ, and AM. Data analysis was performed by SG, MB, and RP, Data interpretation was carried out by SG, MB, RP, and AM. The manuscript was drafted by SG. The content of the manuscript was revised by SG, RP, MB-R, BF, MW, SB, EP, MW, EM-T, MB-M, AZ, and AM. All authors approved the final version of the manuscript.

## Ethical approval

The study protocol was approved by the Bioethics Committee of the Medical University of Silesia (PCN/CBN/0022/KB1/137/I/21/22, date: February 8, 2022) and received favorable opinions from the local bioethics committees of all participating centers.
